# Prevalence and Predictors of Mechanical Restraint in Involuntary Patients in an Open-Door Inpatient Psychiatric Unit

**DOI:** 10.62641/aep.v54i4.2328

**Published:** 2026-08-15

**Authors:** Aurea Fernandez-Ribas, Beltran Jimenez-Fernandez, Manuel Vasquez, Laura Vargas-Puertolas, Encarnacion Romero-Diaz, Marina Delgado-Mari, Laura Miro-Mezquita, Jorge Cuevas-Esteban

**Affiliations:** ^1^Department of Psychiatry and Legal Medicine, Autonomous University of Barcelona, 08193 Cerdanyola del Vallès, Spain; ^2^Department of Psychiatry, Germans Trias i Pujol University Hospital, 08916 Badalona, Spain; ^3^Innovation, Health Economics and Digital Transformation Research Group (INEDIT), Germans Trias i Pujol Research Institute (IGTP), 08916 Badalona, Spain; ^4^Germans Trias i Pujol Research Institute (IGTP), 08916 Badalona, Spain; ^5^Centre for Biomedical Research Network in Mental Health (CIBERSAM), 28029 Madrid, Spain

**Keywords:** mechanical restraint, involuntary admission, open-door policy, schizophrenia, coercive measures

## Abstract

**Background:**

Mechanical restraint (MR) remains one of the most controversial interventions in psychiatry, particularly in involuntarily admitted patients. This study examined the prevalence and clinical predictors of MR in an open-door psychiatric unit in Spain, a context where national MR rates remain among the highest in Europe.

**Methods:**

A retrospective descriptive study of all involuntarily admitted patients (n = 81) was conducted in a 20-bed open-door acute psychiatric unit between June and December 2024. Sociodemographic, diagnostic, and treatment variables were extracted from electronic health records. Bivariate analyses based on exact tests and Firth penalized-likelihood logistic regression were used to identify independent correlates of MR.

**Results:**

MR was applied in 14.8% of patients. In Firth penalized-likelihood multivariable analysis, schizophrenia was the strongest independent correlate of MR (odds ratio [OR] = 9.58, 95% confidence interval [CI]: 1.71–65.89, *p* = 0.010). Male gender showed a significant bivariate association but lost significance after adjustment. MR co-occurred with intramuscular psychotropic medication in 91.7% of cases.

**Conclusions:**

Within this single-centre, exclusively involuntary cohort, care delivered in an open-door setting with structured de-escalation interventions was associated with a comparatively low rate of MR. Schizophrenia emerged as the strongest correlate of MR and may represent a priority target for prevention strategies. The results align with convergent evidence from German, Swiss, and Norwegian studies supporting the safety and effectiveness of open-door approaches, and provide the first Spanish data on MR in this model of care. These findings are preliminary and require confirmation in larger, multicentre samples.

## Introduction

Mechanical restraint (MR) is one of the most contentious and ethically challenging interventions in modern psychiatry, particularly when applied to involuntarily admitted patients [1, 2]. Defined as the use of physical devices to restrict a person’s movement, MR is intended as a last-resort safety measure during acute behavioral crises [1, 2]. However, it is associated with substantial risks, including physical injury, psychological trauma, erosion of therapeutic relationships, and violation of human rights principles [1, 2, 3]. For involuntary patients—already experiencing loss of liberty—MR represents a “double coercion” with profound ethical implications [3].

Reported prevalence of MR in adult psychiatric inpatient settings varies widely internationally, from less than 1% to 51%, largely reflecting differences in national and regional protocols rather than true differences in clinical need [4, 5]. In the multicentre European Evaluation of Coercion in Psychiatry and Harmonization of Best Clinical Practice (EUNOMIA) study of involuntarily admitted patients across ten European countries, the overall frequency of coercive measures (restraint, seclusion, or forced medication) was 38%, with substantial between-country variation [6]; this figure refers to combined coercive measures in involuntary patients and does not represent a national prevalence of MR. In Spanish acute units, published figures range from approximately 12% to 26% [7, 8, 9, 10], and no contemporary standardised national estimate exists. In the Spanish context, several studies provide essential benchmarks. Pérez-Revuelta *et al*. [7] conducted an eight-year retrospective analysis in an acute psychiatric hospitalisation unit, reporting that approximately 12% of admissions involved at least one episode of MR, with involuntary admission and personality disorder among the factors most strongly associated with its use. More recently, Cañabate Ros *et al*. [8] reported strong associations between MR and involuntary admission (odds ratio (OR) = 17.90), previous restraint episodes (OR = 27.69), and the presence of positive psychotic symptoms (OR = 1.35). In our own healthcare region, El-Abidi *et al*. [9] found a 25.6% MR prevalence in a mixed voluntary/involuntary acute unit, further emphasizing the need for reduction strategies in routine clinical practice. Cañabate Ros *et al*. [8] have recently provided further evidence on the sociodemographic and clinical profile of restrained patients in Spain, and comparable prevalence figures have been reported in other Southern European settings [10].

Open-door psychiatric units, increasingly implemented across Europe, aim to enhance autonomy and recovery by removing locked doors and allowing supervised mobility [11, 12]. Large-scale studies—such as Schneeberger *et al*. [13] in Germany—have shown significant reductions in seclusion and restraint in open-door hospitals compared to locked wards without increases in aggressive incidents. More recently, Indregard *et al*. [14] conducted the first randomized controlled trial in Norway, reporting lower coercion prevalence in open-door wards (26.5%) versus treatment-as-usual (33.4%).

Despite the World Health Organization (WHO) Quality Rights initiative and the recent WHO/Office of the United Nations High Commissioner for Human Rights (OHCHR) guidance on mental health, human rights and legislation, which calls for the elimination of coercive practices as part of a global shift toward rights-based mental health care [15, 16], few studies have examined MR prevalence and predictors specifically in open-door settings with exclusively involuntary populations.

To our knowledge, this is the first study to examine the prevalence and predictors of MR in an exclusively involuntary cohort treated in an open-door psychiatric unit in Spain. Existing Spanish data derive from closed-door or mixed-policy settings, and the available open-door evidence comes from German, Swiss, and Norwegian samples combining voluntary and involuntary admissions, in which low coercion rates may partly reflect the lower baseline risk of voluntary patients. By restricting the analysis to involuntarily admitted patients— an exposure repeatedly associated with MR or coercive practices in previous studies [7, 8, 17]—the present study offers a stringent test of the open-door model under a distinct legal and organizational framework, and a national benchmark for future coercion-reduction initiatives.

This study aims to determine the prevalence and identify independent predictors of MR in an exclusively involuntary cohort admitted to an open-door psychiatric unit in Spain. The findings may inform targeted interventions to reduce coercion while maintaining safety, aligning clinical practice with human rights-based mental health care.

## Methods

### Study Design and Setting

This was a retrospective observational chart-review study. The study population comprised all involuntarily admitted patients treated in the acute psychiatric inpatient unit between June and December 2024. This period refers to the clinical admission/treatment period of the cohort. No patients were prospectively enrolled or contacted for research purposes; all variables were extracted retrospectively from routinely collected electronic health records. This retrospective descriptive study was conducted in the acute open-door psychiatric unit of Germans Trias i Pujol University Hospital (GTUH), a general multispecialty teaching hospital affiliated with the Autonomous University of Barcelona. Located in Badalona (Barcelona, Spain), GTUH serves a catchment area of 350,530 adults across four community mental health zones within the North Barcelona health district.

At the time of the study, the psychiatric unit operated as a single 20-bed ward, staffed by a multidisciplinary team including psychiatrists, psychiatry residents, nurses, psychologists, social workers, occupational therapists, and auxiliary personnel. The unit provides intensive treatment for severe mental disorders, integrating pharmacotherapy, psychotherapy, occupational therapy, and social interventions. Since 2019, the Safewards care model—comprising ten structured interventions aimed at reducing conflict and containment—has been progressively implemented throughout the unit [18]. Electroconvulsive therapy was available when clinically indicated.

The unit follows a comprehensive open-door policy, allowing patients up to six hours of supervised daily leave from the ward. Approximately 90–95% of admissions occur through the emergency department, with most patients presenting with acute psychiatric symptoms and/or significant risk of self-harm or harm to others. Roughly 40% of admissions are involuntary (court-mandated per Spanish mental health legislation), while 60% are voluntary.

### Sample and Data Collection

The study population comprised all patients admitted involuntarily to the psychiatric unit during the six-month study period. A total of 81 patients met the inclusion criteria. Data were collected retrospectively from electronic medical records and hospital administrative databases, ensuring comprehensive capture of relevant clinical information. There were no missing data for any of the variables analyzed.

Variables collected included sociodemographic characteristics (age, gender, living arrangements), clinical diagnoses according to Diagnostic and Statistical Manual of Mental Disorders, Fifth Edition (DSM-5) criteria, and treatment-related factors (use of intramuscular medication, application of MR, substance use at admission and discharge, and history of psychiatric admissions). All diagnostic assignments were made by qualified psychiatrists and verified through chart review.

### Variables and Definitions

The primary outcome variable was the use of MR during hospitalization. MR was operationally defined as any use of physical devices to restrict a patient’s movement [1, 4], documented in clinical records according to institutional protocols. This included restraint episodes initiated prior to ward admission (e.g., in the emergency department), provided they were documented in the medical record.

Independent variables included gender, age, living situation, primary psychiatric diagnosis, history of substance use disorders, administration of intramuscular psychotropic medication, and whether the admission was the patient’s first psychiatric hospitalization. Diagnoses were grouped following the DSM-5 chapter structure [19] into psychotic disorders (schizophrenia, schizoaffective disorder, and other psychotic disorders, including unspecified psychotic disorder, delusional disorder, and substance/medication-induced psychotic disorder), affective disorders (bipolar and related disorders, depressive disorders, and unspecified mood disorder), and other disorders. Given prior evidence of their strong association with coercive measures, schizophrenia spectrum disorders were analyzed separately.

### Data Analysis

Descriptive statistics were used to summarize patient characteristics and to calculate the overall prevalence of MR. The distribution of continuous variables was assessed using the Shapiro–Wilk test and visual inspection of histograms and Q–Q plots. As age did not follow a normal distribution (Shapiro–Wilk, *p* = 0.002), continuous variables are presented as median (interquartile range, IQR) and compared between groups with the Mann–Whitney U test. Categorical variables are summarised as counts and percentages and compared using Fisher’s exact test, which is preferred over the Pearson chi-square test when expected cell frequencies are low (notably in 2 × 2 tables), and the Fisher–Freeman–Halton exact test for larger contingency tables, given that more than 20% of cells had expected frequencies below 5.

Variables associated with MR at *p* < 0.10 in bivariate analyses, together with variables of established clinical relevance (age, sex), were considered for the multivariable model. Intramuscular medication was not entered as an independent predictor because it constitutes a concomitant containment intervention applied during the same agitation episode rather than an antecedent patient characteristic, and because its near-complete overlap with the outcome (11 of 12 restrained patients) produced quasi-complete separation; a sensitivity analysis forcing this variable into the model is provided in **Supplementary Table 1**. The final model included sex, age, and schizophrenia. Given the limited number of outcome events (n = 12), the multivariable analysis was performed using Firth’s penalized-likelihood logistic regression, which reduces small-sample bias and yields stable estimates under sparse-data conditions; 95% confidence intervals (CIs) were obtained by profile penalized likelihood, and ORs with 95% CIs and exact *p*-values are reported for each predictor. The multivariable analysis was considered exploratory given the low events-per-variable ratio. Statistical significance was set at two-sided *p* < 0.05. Analyses were conducted using IBM SPSS Statistics version 28.0 (IBM Corp., Armonk, NY, USA); Firth penalized-likelihood logistic regression models were fitted in R version 4.4.3 (R Foundation for Statistical Computing, Vienna, Austria) using the logistf package version 1.26.0.

## Results

### Demographic and Clinical Characteristics

A total of 81 involuntarily admitted patients were included in the analysis. The sample was nearly gender-balanced (49.4% female), with a median age of 42.0 years (IQR: 29–59; range: 18–87 years). MR was applied in 14.8% of cases (n = 12).

As shown in Table 1, male sex was significantly associated with MR (83.3% in the restrained group vs. 44.9% in the non-restrained group; *p* = 0.026.

**Table 1.** **Demographic and clinical characteristics by mechanical restraint status**.

Variable		No restraint (n = 69)	Mechanical restraint (n = 12)	Total (n = 81)	*p*-value
Sex, n (%)					0.026
	Female	38 (55.1%)	2 (16.7%)	40 (49.4%)	
	Male	31 (44.9%)	10 (83.3%)	41 (50.6%)	
Age, median (IQR)		43.0 (34–60)	28.5 (27–46)	42.0 (29–59)	0.100
Living arrangements, n (%)					0.102
	Family	40 (58.0%)	3 (25.0%)	43 (53.1%)	
	Alone	15 (21.7%)	4 (33.3%)	19 (23.5%)	
	Partner	7 (10.1%)	2 (16.7%)	9 (11.1%)	
	Other	7 (10.1%)	3 (25.0%)	10 (12.3%)	
Substance use at admission, n (%)					0.307
	No	51 (73.9%)	7 (58.3%)	58 (71.6%)	
	Yes	18 (26.1%)	5 (41.7%)	23 (28.4%)	
First admission, n (%)					0.760
	No	42 (60.9%)	8 (66.7%)	50 (61.7%)	
	Yes	27 (39.1%)	4 (33.3%)	31 (38.3%)	

Fisher’s exact test (2 × 2 tables) or Fisher–Freeman–Halton exact test (living arrangements); Mann–Whitney U test for age. IQR, interquartile range.

No significant differences were found between restrained and non-restrained patients with respect to age (median 43.0 vs. 28.5 years; *p* = 0.100), living arrangements, substance use at admission, or first psychiatric admission status.

### Diagnostic and Treatment Variables

As displayed in Table 2, broader diagnostic categories (psychotic, affective, or other disorders) were not significantly associated with MR use (*p* = 0.286). However, patients with a diagnosis of schizophrenia had significantly higher odds of being mechanically restrained (*p* = 0.004).

**Table 2.** **Diagnostic and treatment variables by mechanical restraint status**.

Variable		No restraint (n = 69)	Mechanical restraint (n = 12)	Total (n = 81)	*p*-value^1^
Primary diagnosis (DSM-5), n (%)					0.076
Psychotic disorders					
	Schizophrenia	2 (2.9%)	4 (33.3%)	6 (7.4%)	
	Schizoaffective disorder	8 (11.6%)	1 (8.3%)	9 (11.1%)	
	Other psychotic disorders^2^	24 (34.8%)	4 (33.3%)	28 (34.6%)	
Affective disorders					
	Bipolar and related disorders	16 (23.2%)	2 (16.7%)	18 (22.2%)	
	Depressive disorders	10 (14.5%)	1 (8.3%)	11 (13.6%)	
	Unspecified mood disorder	3 (4.3%)	0 (0.0%)	3 (3.7%)	
	Other disorders^3^	6 (8.7%)	0 (0.0%)	6 (7.4%)	
Schizophrenia, n (%)					0.004
	No	67 (97.1%)	8 (66.7%)	75 (92.6%)	
	Yes	2 (2.9%)	4 (33.3%)	6 (7.4%)	
Diagnostic category, n (%)					0.286
	Affective disorders	29 (42.0%)	3 (25.0%)	32 (39.5%)	
	Psychotic disorders	34 (49.3%)	9 (75.0%)	43 (53.1%)	
	Other disorders	6 (8.7%)	0 (0.0%)	6 (7.4%)	
Intramuscular medication, n (%)					<0.001
	No	67 (97.1%)	1 (8.3%)	68 (84.0%)	
	Yes	2 (2.9%)	11 (91.7%)	13 (16.0%)	
Substance use disorder at discharge, n (%)					0.140
	No	55 (79.7%)	7 (58.3%)	62 (76.5%)	
	Yes	14 (20.3%)	5 (41.7%)	19 (23.5%)	

^1^Fisher’s exact test (2×2 tables) or Fisher–Freeman–Halton exact test (Primary Diagnosis, Diagnostic Category). ^2^Includes unspecified psychotic disorder, delusional disorder, and substance/medication-induced psychotic disorder. ^3^Includes gambling disorder (n = 1), anorexia nervosa (n = 1), autism spectrum disorder (n = 1), major neurocognitive disorder (n = 2), and fetal alcohol syndrome (n = 1). Classification based on DSM-5 [19]. DSM-5, Diagnostic and Statistical Manual of Mental Disorders, Fifth Edition.

The use of intramuscular psychotropic medication was strongly associated with MR. Among restrained patients, 91.7% received intramuscular medication compared to 2.9% of non-restrained patients (*p* < 0.001).

Substance use disorder at discharge showed no statistically significant association with MR (*p* = 0.140).

### Multivariate Analysis

As shown in Table 3, schizophrenia was the only statistically significant independent correlate of MR after adjustment for age and sex (OR = 9.58; 95% CI: 1.71–65.89; *p* = 0.010).

**Table 3.** **Firth penalized-likelihood logistic regression predicting mechanical restraint use**.

Variable	B	SE	χ^2^	df	*p*-value	OR (Exp B)	95% CI for OR
Male sex	1.230	0.780	2.81	1	0.094	3.42	0.82–19.51
Age	–0.019	0.021	0.87	1	0.351	0.98	0.94–1.02
Schizophrenia	2.260	0.963	6.58	1	0.010	9.58	1.71–65.89
Constant	–1.917	1.144	—	—	—	—	—

Firth penalized-likelihood estimation; χ^2^ and *p*-values from penalized-likelihood-ratio tests; 95% confidence intervals (CI) by profile penalized likelihood. Odds ratio (OR) is not reported for the constant term. SE, standard error.

Although male sex was significantly associated with MR in bivariate analysis, it did not retain statistical significance in the multivariable model (OR = 3.42; 95% CI: 0.82–19.51; *p* = 0.094). Age was not independently associated with MR (OR = 0.98; 95% CI: 0.94–1.02; *p* = 0.351). A sensitivity analysis forcing intramuscular medication into the model (**Supplementary Table 1**) produced extreme and unstable estimates (OR = 1255.5; 95% CI: 39.8–6.8×10^6^), consistent with quasi-complete separation, supporting its exclusion from the primary model.

## Discussion

This study identified a 14.8% prevalence of MR among involuntarily admitted patients in an open-door acute psychiatric unit. This finding is particularly relevant given that involuntary admission is one of the strongest predictors of MR use across psychiatric settings. By focusing exclusively on this high-risk subgroup, the study provides a stringent test of restraint-reduction strategies within the Spanish healthcare system.

As a benchmark, the multicentre EUNOMIA study reported a 38% frequency of coercive measures among involuntarily admitted patients across ten European countries [6]; our 14.8% rate is markedly lower, although this comparison must be interpreted with caution, since that figure aggregates restraint, seclusion, and forced medication across multiple countries rather than MR in Spain specifically. Within the Spanish literature, our rate falls at the lower end of the range reported in acute units (approximately 12–26%) [7, 8, 9, 10]. Higher restrained proportion (50 of 91 patients) has been reported by Cañabate Ros *et al*. [8]; however, that figure derives from a purposive sample that deliberately included comparable numbers of restrained and non-restrained patients and does not represent a population prevalence—as the authors themselves acknowledge. Rather than a single national benchmark—which does not currently exist for MR in Spain—these comparisons should be read in light of differing case-mix, sampling designs, denominators, and local protocols [4]. These comparisons suggest that open-door policies—especially when combined with structured interventions such as Safewards [18]—may contribute to reducing coercive practices in psychiatric care.

Internationally, our findings align with the growing body of evidence supporting the effectiveness of open-door models. The 10-year longitudinal study by Krückl *et al*. [20] and the multicenter analysis by Schneeberger *et al*. [13] both reported significant reductions in the use of seclusion and restraint in hospitals with open-door policies. Indregard *et al*. [14] also demonstrated that patients treated in open-door wards had lower MR rates compared to treatment-as-usual wards in a randomized controlled trial. Although direct comparisons are limited by differences in legal frameworks, population characteristics, and definitions of coercion, the convergence of results across healthcare systems strengthens the argument that architectural and policy-level changes can lead to meaningful reductions in coercion without compromising safety.

Furthermore, these international studies underscore the importance of sustained institutional commitment and long-term implementation. In the study by Schneeberger *et al*. [13], the benefits of open-door policies were observed over more than a decade and across hundreds of thousands of admissions, highlighting the scalability and sustainability of this approach Similarly, Krückl *et al*. [20] demonstrated that patients initially admitted to open wards but later transferred to closed settings had a significantly higher likelihood of being subjected to coercive measures, underlining the potential value of maintaining treatment continuity in open settings.

A key clinical finding of this study is the strong association between schizophrenia diagnosis and MR use (OR = 9.58). This diagnostic group accounted for one-third of all restrained patients, despite representing less than 10% of the total sample. This is consistent with evidence identifying male sex, younger age, and acute psychotic presentations among the factors most consistently associated with coercive measures [4, 6, 20], although a recent systematic review emphasises that risk is multifactorial and that no single factor is firmly established [17] The width of the confidence interval for this association, which persists after bias-corrected estimation, reflects the small number of patients with schizophrenia in the cohort (n = 6); the magnitude of the effect should therefore be interpreted as preliminary, although its direction and statistical significance were robust across estimation methods. The strength of this association underlines the need for diagnosis-specific preventive strategies, including early identification of agitation, specialized de-escalation protocols, and targeted psychosocial support from admission onwards. Notably, in a cluster-randomized controlled trial conducted in high-security wards for men with schizophrenia or psychotic illness and violent behavior, seclusion and restraint were substantially reduced without a corresponding increase in violence through a structured, multicomponent intervention based on seclusion-restraint prevention strategies [21].

Another important observation was the high co-occurrence between MR and intramuscular medication administration (91.7%). This suggests that these interventions are often deployed together as part of crisis management protocols. While potentially necessary in acute situations, this practice raises important questions about the availability and effectiveness of alternative de-escalation techniques, particularly because the use of multiple, combined coercive measures has been associated with greater subjective distress and more physical adverse effects than single interventions [22]. Future quality improvement initiatives should explore whether early intervention and structured verbal de-escalation strategies can reduce the reliance on combined physical and pharmacological containment. In the Spanish context, a regional multicomponent programme based on the Six Core Strategies significantly reduced MR hours across acute psychiatric wards [23].

### Limitations

This study has several limitations. First, its retrospective design limits the ability to capture contextual and dynamic factors influencing clinical decision-making. Second, we did not collect data on episodes of psychomotor agitation that were successfully managed without MR. This restricts our ability to identify protective factors or effective de-escalation practices, and may underestimate the broader clinical management of agitation. Third, the sample was drawn from a single center, potentially limiting generalizability to other psychiatric services, particularly those operating under different legal or organizational conditions. Finally, the small sample size may have limited statistical power to detect other relevant predictors. In particular, with only 12 restraint events, the events-per-variable ratio of the multivariable model was low. Although Firth’s penalized-likelihood estimation was used to mitigate small-sample bias, the wide confidence intervals—particularly for schizophrenia—indicate limited precision, and the multivariable results should be regarded as exploratory and hypothesis-generating. Replication in larger, multicentre samples is required.

## Conclusions

Our findings suggest that comparatively low rates of MR are achievable even among high-risk involuntary psychiatric inpatients when care is delivered in an open-door setting with structured therapeutic interventions; however, given the observational, single-centre design, this association should be interpreted as hypothesis-generating rather than as evidence of effectiveness. The strong association between schizophrenia and MR highlights the importance of developing targeted preventive strategies for this group. While architectural and policy changes appear necessary, they are not sufficient alone; sustained investment in staff training, structured de-escalation, and diagnosis-specific interventions remains essential. These results support the continued shift toward rights-based, non-coercive psychiatric care in line with international human rights frameworks.

## Availability of Data and Materials

Data are not publicly available due to patient confidentiality restrictions. De-identified data supporting the findings of this study are available from the corresponding author upon reasonable request and subject to institutional data governance approval.

## Supplementary Material

Supplementary material associated with this article can be found, in the online version, at https://doi.org/10.62641/aep.v54i4.2328.
