# Supplementary material for: Prevalence and Predictors of Mechanical Restraint in Involuntary Patients in an Open-Door Inpatient Psychiatric Unit
Source: Actas Esp Psiquiatr. 2026 Aug 15;54(4):963–70. doi: 10.62641/aep.v54i4.2328 (PMC13571176; doi:10.62641/aep.v54i4.2328)
Supplement: Supplementary file 1 [file ActEsp-54-4-963-970-s1.zip › ActEsp-54-4-963-970.docx]

**Supplementary Material**

*Prevalence and Predictors of Mechanical Restraint in Involuntary Patients in an Open-Door Inpatient Psychiatric Unit*

**Supplementary Table 1. Sensitivity Analysis: Firth Penalized-Likelihood Logistic Regression Predicting Mechanical Restraint, Forcing Intramuscular Medication Into the Model**

| **Variable** | **B** | **SE** | **p-value** | **OR** | **95% CI for OR** |
| --- | --- | --- | --- | --- | --- |
| Male sex | 3.829 | 1.869 | 0.015 | 46.00 | 1.84 – 8785.3 |
| Age | 0.065 | 0.041 | 0.098 | 1.07 | 0.99 – 1.26 |
| Schizophrenia | −0.521 | 2.203 | 0.843 | 0.59 | 0.002 – 2332.0 |
| Intramuscular medication | 7.135 | 2.191 | <0.001 | 1255.5 | 39.8 – 6.8×10⁶ |
| Constant | −10.269 | 3.901 | — | — | — |

*Note. Standard maximum-likelihood estimation did not converge due to quasi-complete separation (intramuscular medication: 11/12 restrained vs. 2/69 non-restrained patients exposed). Firth penalized-likelihood estimates are shown; p-values from penalized-likelihood-ratio tests; 95% confidence intervals (CI) by profile penalized likelihood. The extreme magnitude and width of the estimates indicate model degeneracy: intramuscular medication is a concomitant containment intervention applied during the same agitation episode as mechanical restraint, not an antecedent predictor. This analysis is presented solely to document the basis for its exclusion from the primary model (see Methods, Data Analysis). OR is not reported for the constant term.*
